# Introducing the non-B DNA Motif Search Tool (nBMST)

**DOI:** 10.1186/gb-2011-12-s1-p34

**Published:** 2011-09-19

**Authors:** Regina Z Cer, Kevin H Bruce, Duncan E Donohue, Alpay N Temiz, Albino Bacolla, Uma S Mudunuri, Ming Yi, Natalia Volfovsky, Brian T Luke, Jack R Collins, Robert M Stephens

**Affiliations:** 1 Advanced Biomedical Computing Center, Information Systems Program, SAIC-Frederick, National Cancer Institute-Frederick, Frederick, MD 21702, USA; 2 The Dell Pediatric Research Institute, Department of Pharmacy, The University of Texas at Austin, 1400 Barbara Jordan Boulevard, Austin, TX 78723, USA

## 

DNA sequence motifs with the ability to form non-B (non-canonical) structures have been linked to a variety of regulatory and pathological processes. Although the exact mechanism is unknown, recent work has provided significant evidence that non-B DNA structures may play a role in DNA instability and mutagenesis, leading to both DNA rearrangements and increased mutational rates, which are hallmarks of cancer. We have developed algorithms to identify a wide variety of non-B-DNA-forming motifs, including G-quadruplex-forming repeats, direct repeats and slipped motifs, inverted repeats and cruciform motifs, mirror repeats and triplex motifs, and A-phased repeats. After identifying these motifs in the mammalian reference genomes of human, mouse, chimpanzee, macaque, cow, dog, rat and platypus, the data were made publicly available in non-B DB [1]. However, it soon became apparent that it was not feasible to annotate the ever-growing list of genomic data and that it would be more effective to provide researchers with a systematic tool to predict these motifs in their own genomic data. Thus, the non-B DNA Motif Search Tool (nBMST) was created, and it is freely available online [2]. nBMST is a web interface that enables researchers to interactively submit any DNA sequence for searching for non-B DNA motifs. Once a user submits one or more DNA sequences in FASTA format, nBMST returns a comprehensive results page that contains the following: downloadable files in both a tab-delimited format and a generic feature format (GFF); a visualization, including PNG images; and a dynamic genome browser created using the Generic Genome Browser (GBrowse) [3] (version 2.0). Currently, nBMST allows file sizes of up to 20 MB of DNA sequence to be uploaded and stores the results for registered users for up to six months. In summary, the purpose of nBMST is to help provide insight into the involvement of alternative DNA conformations in cancer and other diseases, as well as into other potential biological functions.
